# Size-Dependent Phonon-Assisted Anti-Stokes Photoluminescence in Nanocrystals of Organometal Perovskites

**DOI:** 10.3390/nano12183184

**Published:** 2022-09-14

**Authors:** Kairolla Sekerbayev, Yerzhan Taurbayev, Gauhar Mussabek, Saule Baktygerey, Nikolay S. Pokryshkin, Valery G. Yakunin, Zhandos Utegulov, Victor Yu. Timoshenko

**Affiliations:** 1 Institute of Experimental and Theoretical Physics, Al-Farabi Kazakh National University, Almaty 050040, Kazakhstan; 2 Department of Physics, School of Sciences and Humanities, Nazarbayev University, Nur-Sultan 010000, Kazakhstan; 3 Institute of Information and Computational Technologies, 125, Pushkin Str., Almaty 050000, Kazakhstan; 4 Phys-Bio Institute, University “MEPhI”, 115409 Moscow, Russia; 5 Faculty of Physics, Lomonosov Moscow State University, 119991 Moscow, Russia

**Keywords:** photoluminescence, anti-stokes, perovskites, methylammonium lead bromide, nanocrystals, phonons

## Abstract

Anti-Stokes photoluminescence (ASPL), which is an up-conversion phonon-assisted process of the radiative recombination of photoexcited charge carriers, was investigated in methylammonium lead bromide (MALB) perovskite nanocrystals (NCs) with mean sizes that varied from about 6 to 120 nm. The structure properties of the MALB NCs were investigated by means of the scanning and transmission electron microscopy, X-ray diffraction and Raman spectroscopy. ASPL spectra of MALB NCs were measured under near-resonant laser excitation with a photon energy of 2.33 eV and they were compared with the results of the photoluminescence (PL) measurements under non-resonant excitation at 3.06 eV to reveal a contribution of phonon-assisted processes in ASPL. MALB NCs with a mean size of about 6 nm were found to demonstrate the most efficient ASPL, which is explained by an enhanced contribution of the phonon absorption process during the photoexcitation of small NCs. The obtained results can be useful for the application of nanocrystalline organometal perovskites in optoelectronic and all-optical solid-state cooling devices.

## 1. Introduction

It is known that materials with efficient phonon-assisted up-conversion photoluminescence (PL) have potential applications in the field of the optical cooling of condensed phase materials. The conversion of absorbed low-energy photons into high-energy photons by a medium is called the light up-conversion process. This up-conversion process is also called anti-Stokes photoluminescence (ASPL), which has been observed in various systems spanning from atoms and molecules [1,2] to polymers [3,4], rare-earth doped materials [5,6], organic dyes [7,8], carbon nanotubes [9], II-VI semiconducting nanobelts [10,11] and colloidal semiconductor nanocrystals (NCs) [12,13,14]. Up-conversion optical processes have many applications including multi-color displays [15], dynamical imaging microscopy [16], bio-imaging systems [17,18], unconventional lasers [19] and solid-state optical refrigeration devices [20,21].

Due to their direct bandgap and large optical absorption, lead halide perovskite NCs exhibit efficient PL with a high quantum yield (QY) which makes them an attractive optoelectronic material [22]. NCs and films of organometal perovskites as methylammonium lead halide (CH_3_NH_3_PbX_3_, where X = I, Br, Cl) have been explored for applications in such optoelectronics devices as solar cells, photodetectors, light-emitting devices and lasers [23,24,25]. A high QY is also required for the optical cooling application of perovskites [20,26]. The ASPL process in perovskite NCs has been broadly studied over the past decade [27,28]. ASPL was demonstrated in the NCs of all-inorganic perovskite as CsPbX_3_ [28,29] and in organometal ones [30]. The optical cooling effect was investigated for both the bulk and nanostructured perovskites [31,32]. The optical cooling in relatively large perovskite NCs with sizes comparable with the optical wavelength can be enhanced by coupling with Mie resonances [33]. The efficiency of ASPL optical cooling can be improved by the size-tunable control of the absorption and the PL band of perovskite NCs due to the quantum confinement [31]. Additionally, colloidal solutions and films of perovskite NCs can be prepared by the inexpensive wet chemistry approach [34]. To the best of our knowledge, size-dependent ASPL in methylammonium lead bromide (MALB) has been insufficiently studied before, while such studies can be useful to assess the potential of MALB NC for optical cooling and other photonic applications.

In this work, the size-dependent ASPL efficiency in MALB perovskite NCs excited by photons with energies within the PL band is investigated. Sample PL properties are also probed by excitations with photon energies exceeding the PL band. The obtained results indicate a difference in ASPL processes in large and small NCs. In small NCs, enhanced phonon-assisted light absorption promotes ASPL excitation.

## 2. Materials and Methods

Methylammonium bromide (CH_3_NH_3_Br), lead bromide (PbBr_2_), octylamine, dimethylformamide, toluene, oleic acid and benzyl alcohol were acquired from Sigma-Aldrich (Sigma-Aldrich Chemie GmbH, Taufkirchen, Germany).

MALB nanocrystals were synthesized by the ligand-assisted reprecipitation (LARP) technique [35]. In this colloidal chemistry method, the perovskite precursors dissolved in “pro-solvent” were added into “anti-solvent” containing organic ligands such as octylamine, oleic acid and benzyl alcohol [36]. The “anti-solvent” typically triggers perovskite crystallization, while the organic ligands impede the crystal growth. According to this method, 22.4 mg of CH_3_NH_3_Br and 73.4 mg of PbBr_2_ were dissolved in 1 mL of dimethylformamide, resulting in a solvent solution containing MALB perovskite components. The “anti-solvent” solution consists of 5 mL of toluene, 10–30 μL of octylamine, 1 mL of oleic acid and 1 mL of benzyl alcohol. NC size was controlled by the octylamine concertation, which was changed from 0.2 to 0.6%. A total of 150 μL of perovskite solution was injected into this “anti-solvent” solution and was stirred for 5 min. The obtained NC suspension was centrifuged for 10 min at 6000 rpm, which formed a supernatant (the top part of the solution) and precipitate. The supernatant was discarded, and the precipitate was dispersed in the mixture of 2 mL of toluene and 2 μL of octylamine. The obtained suspension was subsequently centrifuged for 5 min at 8000 rpm. The precipitate was dispersed in 2 mL of toluene. Both the supernatant and precipitate suspensions contained NCs and were used in perovskite NC film preparations. NC thin film for spectroscopic investigations was prepared by the drop casting of the MALB NC suspensions on a quartz substrate followed by drying in air. The film thickness was controlled with the amount of the drop-casted suspension and was about 1 micrometer.

The structural properties and morphology of the MALB NCs were characterized by using a Crossbeam 540 (Carl Zeiss) scanning electron microscope (SEM) and a JEM-1400 Plus (JEOL) transmission electron microscope (TEM) for the samples deposited on an optically polished crystalline silicon wafer and carbon-coated gold grid, respectively. The X-ray diffraction (XRD) patterns were collected by using a Radian-02 X-ray diffractometer with a Cu-Kα radiation source.

A Raman confocal microscope Confotec MR350 (SOL Instruments) with continuum wave (CW) 532 nm laser excitation was used to measure the ASPL and PL. The laser incident intensity was 1 kW/cm^2^. Additionally, non-resonant excitation by LED at 405 nm with an intensity of 0.1 kW/cm^2^ was used. The PL and ASPL spectra were detected with a grating MS 3504i monochromator equipped with an Andor iStar 340T intensified CCD detector. For suppressing 532 nm of excitation light, a Bragg grating notch filter centered at 532 nm (with spectral windows of 4 nm) was placed in the entrance of the monochromator. Non-resonant PL measurements were carried out with the same filter. All the measurements were conducted at room temperature in air.

## 3. Results and Discussion

**Figure 1:**
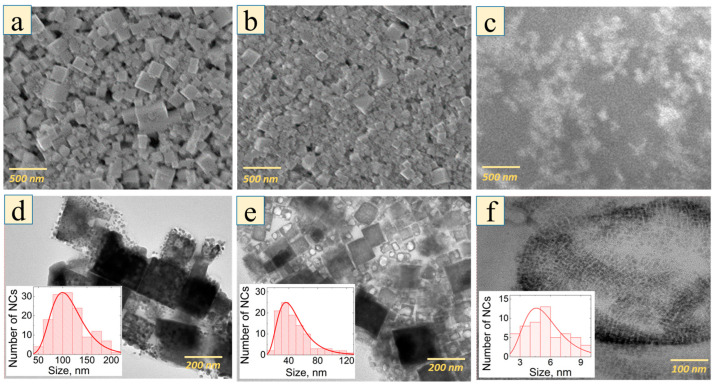
(**a**–**c**) SEM images of MALB perovskite NCs of series L (**a**), M (**b**) and S (**c**); (**d**–**f**) TEM images for the samples of series L (**d**), M (**e**) and S (**f**) with corresponding size distributions shown in insets where red lines are lognormal fits.

According to the SEM and TEM data (Figure 1), the synthesized MALB NCs were characterized by their cubic shape and average lateral sizes of 5.5 ± 1.5, 46 ± 4 and 120 ± 2 nm for the samples obtained with different concentrations of octylamine. The measured size distributions are typical for the perovskite NCs obtained by the LARP technique because of the spontaneous NC growth [35,36]. The larger the concentration of octylamine, the smaller the size of the NCs, as shown in Table 1.

**Figure 2:**
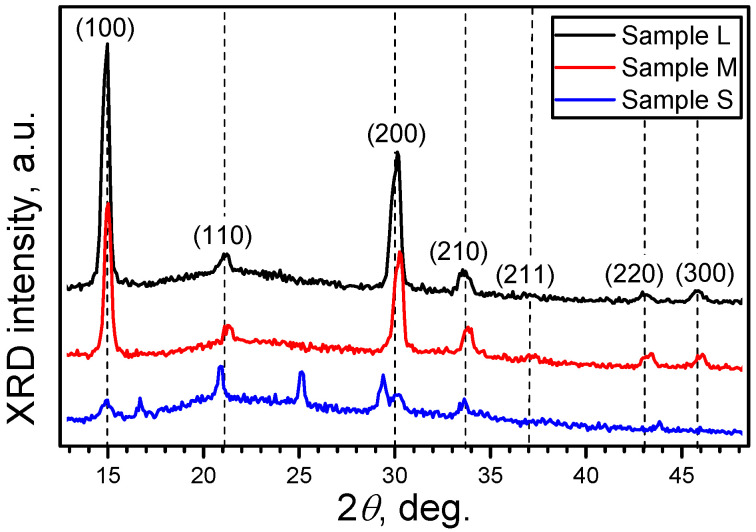
XRD patterns of thin layers of MALB NCs of L series (black line), M (red line) and S (blue line) deposited on quartz substrates.

The obtained XRD patterns (Figure 2) for the samples of the series of M and L demonstrated sharp peaks inherent for the cubic MALB crystals [37]. While the pattern for the NCs of the S series also exhibited features of the (100) and (200) atomic planes of the cubic MALB lattice, the corresponding peaks were noticeably weaker and broadened. The latter was obviously caused by the small NC size. Additional narrow peaks of the XRD signals were also observed in the samples of series M and S at about 17°, 25° and 29°. These angles can be related to the residual perovskite precursor PbBr_2_ [38]. Overlapping with these precursors’ peaks can explain the observed shift in the (110) and (200) peaks in the samples of series M and S. Thus, the XRD analysis confirms that the synthesized samples were predominately composed of the cubic MALB NCs.

**Figure 3:**
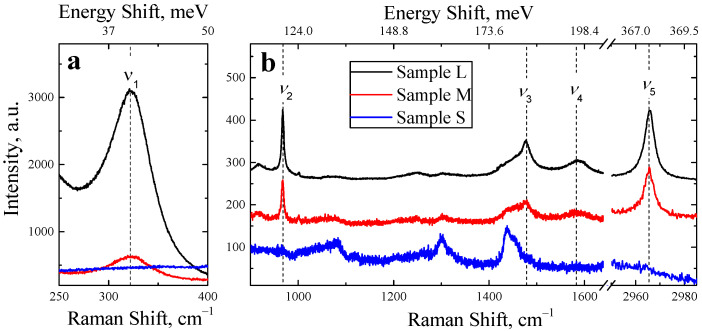
(**a**) Raman spectra of thin films of MALB NCs of L series (black line), M (red line) and S (blue line), 50 nm and 130 nm in the spectral regions of 250–400 cm^–1^; (**b**) Raman spectra of the same samples in the region of 900–3000 cm^–1^. Vertical dashed lines refer to vibration frequencies of the MALB lattice.

Figure 3 shows typical Raman spectra of the prepared MALB NCs of all the series. The spectra consisted of typical lines of MALB perovskites at ν_1_ = 322 cm^–1^, ν_2_ = 967 cm^−1^, ν_3_ = 1478 cm^–1^, ν_4_ = 1581 cm^–1^ and ν_5_ = 2965 cm^–1^, which can be assigned to the MA^+^ rotation, C–N stretching, NH_3_^+^ symmetric deformation, C–N stretching and CH_3_ symmetric stretching modes, respectively, [39] as summarized in Table 2. Besides the Raman lines of MALB, the spectra exhibited lines at 1083, 1301, 1430 and 1655 cm^–1^, which can be related to the vibration modes in oleic acid ligands [40,41]. While the MALB Raman peak intensities decreased while the NC size decreased, the ligand-related peaks became more intensive. This fact is explained by the increased surface-to-bulk ratio in the small MALB NCs that resulted in a larger contribution of the residual ligands.

**Figure 4:**
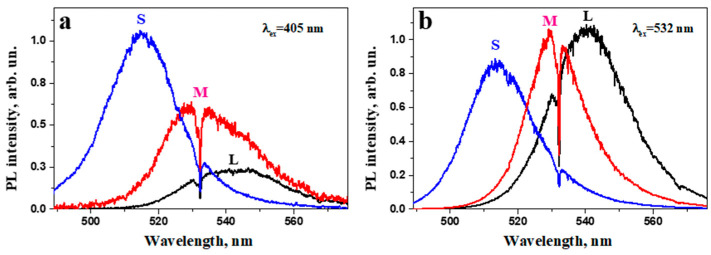
PL spectra for the samples L (black line), M (red line) and S (blue line) excited at 405 nm (**a**) and 532 nm (**b**). The spectral dip at 532 nm was caused by the notch filter.

PL spectra of MALB NCs under 405 and 532 nm laser excitation are shown in Figure 4. One can see that small NCs (S series) showed more intense ASPL emissions in comparison with the NCs of the L series. The sample with 6 nm sized NCs exhibited the PL band shift to the high energy region by ~0.1 eV compared to that for the samples of the L series. This PL band shift to the high energy region is related to the quantum confinement effect due to the small size of the MALB NCs [42]. The observed PL spectra broadening is related to the size distribution of the NCs and the electron–phonon interaction [43].

**Figure 5:**
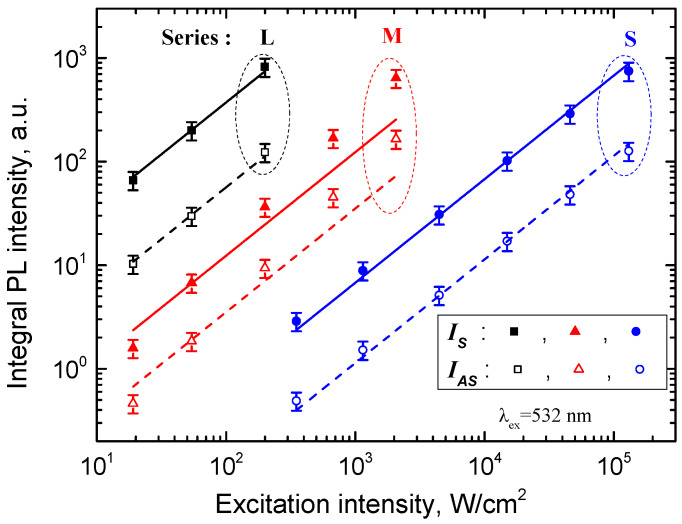
Dependences of the integrated PL intensities of the Stokes (solid symbols) and anti-Stokes (open symbols) parts for series L (black symbols), M (red symbols) and S (blue symbols) on intensity of the excitation at 532 nm. The corresponding linear fits are plotted by solid and dashed lines for the Stokes and anti-Stokes PL intensities, respectively.

Both the anti-Stokes and Stokes parts of MALB NCs PL had a nearly linear dependence on excitation power, as shown in Figure 5. The linear dependence on excitation power indicates a one-photon excitation process and is typical for NCs [12,44]. The observed non-linear rise of the PL intensity of the M series samples at excitation intensity > 1 kW/cm^2^ can be related to a contribution of the bimolecular mechanism of charge carrier recombination in interconnected MALB NCs [42]. This mechanism does not seem to be efficient in smaller NCs because of a larger influence of the surface trapping and an additional enhancement of the Auger recombination due to the breaking the phonon selection rules [43].

**Figure 6:**
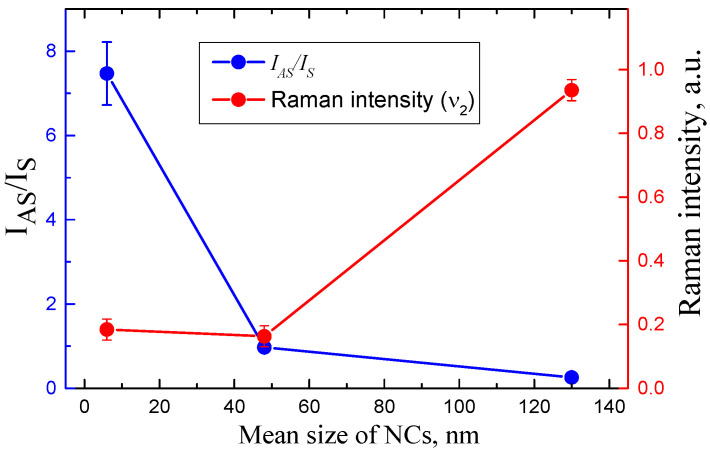
Dependences of the $I_{AS}/I_{S}$ ration and intensity of the Raman signal on mean size of MALB NCs.

Figure 6 shows the ratio of the anti-Stokes and Stokes parts of the PL integral intensities depending on the mean size of MALB NCs. The anti-Stokes and Stokes parts are calculated as follows:

(1) $$I_{AS}=\int_{\lambda_{1}}^{\lambda_{0}}I_{PL}\left(\lambda \right)d\lambda \;,\;\;\;\;I_{S}=\int_{\lambda_{0}}^{\lambda_{2}}I_{PL}\left(\lambda \right)d\lambda$$

where *λ*_1_ = 499 nm, *λ*_0_ = 532 nm and *λ*_2_ = 577 nm, which are chosen according to the PL measurement conditions.

The Stokes part of the PL intensity was larger than the anti-Stokes one for the samples of series L with the largest NCs. The samples of series M with medium sizes of NCs had almost equal the Stokes and anti-Stokes parts of the PL spectra. The anti-Stokes part was almost eight times larger than the Stokes one for the samples of series S with the smallest NCs. The enhancement of ASPL in the latter series was correlated with the decrease in the Raman intensity, as shown in Figure 6.

The phonon-assisted excitation process probability increased in the smaller NCs. This can explain the high ASPL efficiency in the samples of the L series. In small semiconductor NCs, the electron–phonon interaction can be more effective when the selection rules for phonon-assisted optical transitions are violated [45].

When the photon energy in small MALB NCs is insufficient to generate an exciton state, the lacking energy should be provided from phonons in order to realize the ASPL excitation. The simultaneous absorption of one or more phonons followed by photon absorption can be related to the phonon contribution in ASPL. As it is known from the Raman spectroscopy, MALB perovskites vibration modes include: the MA^+^ rotation (ν_1_ =40 meV), C-N (ν_2_ = 120 meV) and NH_3_^+^ symmetric breathing (ν_3_ =183 meV). Under excitation with 2.33 eV, all of these modes can contribute to the exciton generation. In the direct gap semiconductors due to the quasi-momentum conservation, the ASPL process requires two phonons to be absorbed. Therefore, in large MALB NCs, the two-phonon absorption can be the main process of the ASPL excitation. In contrast, in small NCs, the selection rule breaking also allows for optical transitions accompanied with one-phonon absorption. At room temperature and high phonon energy, the latter seems to be more probable than the two-phonon process due to both the temperature-dependent Bose–Einstein statistics, which controls the population of the phonon states. Since the ASPL spectrum of the small MALB NCs corresponds with the exciton energies of 2.43–2.55 eV, which are 100–200 meV above the exciting photon energy, the ASPL excitation can be efficiently realized via the one-phonon absorption for the phonon modes as ν_2,_ ν_3_ and ν_4_ (see Table 2).

**Figure 7:**
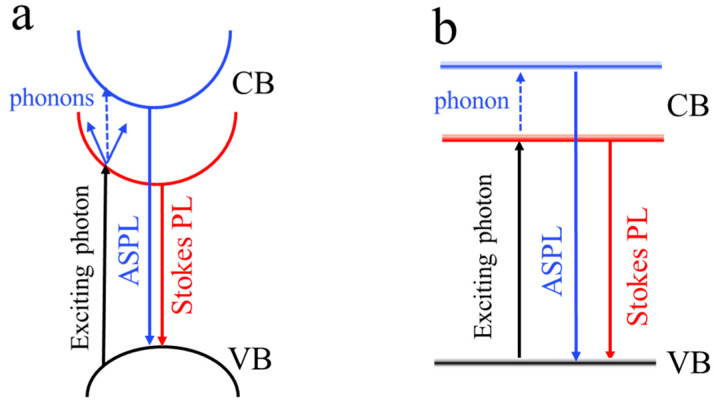
Energy diagrams of the excitation, PL and ASPL in large (**a**) and small (**b**) MALB NCs.

Figure 7a,b shows schematic representations of the energy diagrams for the ASPL excitation and emission in large and small perovskite NCs, respectively. In large nanocrystals, a photon can form an exciton without the contribution of phonons, since its energy is high enough to excite an electron from the valence band to the exciton state. This exciton recombines with the energy nearby the exciting photon one and emits Stokes PL, while ASPL can be excited due to either the simultaneous absorption of two phonons with zero total quasi-momentum or it can be realized in a small NC fraction with exciton energies above the exciting photon one. In small NCs, those band gaps that are below the exciting photon energy of an exciton can be generated only because of an additional energy provided by the phonons, and the one-phonon assisted light absorption is responsible for the ASPL excitation, as shown schematically in Figure 7b.

## 4. Conclusions

Size-dependent phonon-assisted anti-Stokes photoluminescence was observed in MALB perovskite NCs under excitation within the PL band. The observed enhancement of ASPL intensity upon reduction in the mean size of NCs can be explained by the strong electron–phonon interaction promoting the simultaneous absorption of exciting photons and phonons in smaller nanocrystals. Further details of the contribution of phonons to the ASPL process in perovskite NCs can be obtained, for example, by using time-resolved optical measurements.

## Figures and Tables

**Table 1:** Prepared series of MALB NCs with different mean sizes and PL band wavelengths according to the TEM data and PL measurements under non-resonant excitation, respectively.

Series of Samples	Octylamine, %	NCs Mean Size, nm	PL Wavelength, nm
S (small NCs)	0.6	5.5 ± 1.5	515 ± 1
M (medium NCs)	0.4	46.0 ± 4.0	534 ± 1
L (large NCs)	0.2	120 ± 24	543 ± 1

**Table 2:** Vibrational mode assignment for the Raman spectrum of MALB perovskites.

Mode Notation	Frequency, cm^−1^ (meV)	Description
ν_1_	322 (40)	MA rotation
ν_2_	967 (120)	C–N stretching
ν_3_	1478 (183)	NH_3+_ symmetric deformation
ν_4_	1581 (196)	C–N twisting
ν_5_	2965 (368)	CH_3_ symmetric stretching

## Data Availability

Not applicable.
